# The functional highly sensitive brain: a review of the brain circuits underlying sensory processing sensitivity and seemingly related disorders

**DOI:** 10.1098/rstb.2017.0161

**Published:** 2018-02-26

**Authors:** Bianca Acevedo, Elaine Aron, Sarah Pospos, Dana Jessen

**Affiliations:** 1 Neuroscience Research Institute, University of California, Santa Barbara, CA, USA; 2 Department of Psychology, Stony Brook University, Stony Brook, NY, USA

**Keywords:** sensory processing sensitivity, autism spectrum disorders, fMRI, neural systems

## Abstract

During the past decade, research on the biological basis of sensory processing sensitivity (SPS)—a genetically based trait associated with greater sensitivity and responsivity to environmental and social stimuli—has burgeoned. As researchers try to characterize this trait, it is still unclear how SPS is distinct from seemingly related clinical disorders that have overlapping symptoms, such as sensitivity to the environment and hyper-responsiveness to incoming stimuli. Thus, in this review, we compare the neural regions implicated in SPS with those found in fMRI studies of—Autism Spectrum Disorder (ASD), Schizophrenia (SZ) and Post-Traumatic Stress Disorder (PTSD) to elucidate the neural markers and cardinal features of SPS versus these seemingly related clinical disorders. We propose that SPS is a stable trait that is characterized by greater empathy, awareness, responsivity and depth of processing to salient stimuli. We conclude that SPS is distinct from ASD, SZ and PTSD in that in response to social and emotional stimuli, SPS differentially engages brain regions involved in reward processing, memory, physiological homeostasis, self-other processing, empathy and awareness. We suggest that this serves species survival via deep integration and memory for environmental and social information that may subserve well-being and cooperation.

This article is part of the theme issue ‘Diverse perspectives on diversity: multi-disciplinary approaches to taxonomies of individual differences’.

## 1. Introduction

Clinically, sensory processing issues manifest as inappropriate responses to stimuli that involve emotional and behavioural disruptions, and interfere with an individual's daily functioning [1]. As such, ‘sensitivity’ to sensory input may be defined positively as the ability to perceive small changes in stimulus intensity [2], or as a negative reaction to a low-threshold stimulus [3]. Correspondingly, neural ‘hyper’-responsiveness is ‘over-reactivity’ to a stimulus, while ‘hypo’-responsiveness is the absence of a typical response [4,5].

However, a variety of factors affect how individuals express their ‘sensitivity’ to a stimulus, such as their childhood environment, their predisposition to ‘sensitivity’, as well as other individual factors (including co-occurring disorders), and the context in which a particular stimulus occurs. In this review, we will focus on the specific ‘sensitivity’ trait termed ‘sensory processing sensitivity’ (SPS) or ‘high environmental sensitivity’ (ES) as measured by the HSP Scale [6]. Environmental sensitivity is considered a fundamental trait found on a spectrum and is defined as the degree to which an individual may register, process and respond to external factors [7]. Based on the Highly Sensitive Child scale, a reliable self-report measure of SPS or environmental sensitivity, there appear to be three distinct groups of unique levels of environmental sensitivity: low, medium and high, with high sensitivity making up about 20–35% of a UK sample ranging in age from 8–19 years [8]. The three levels of environmental sensitivity are maintained across childhood, adolescence and adulthood, and appear to be comprised of the same neurophysiological and psychological factors but are manifested in varying degrees within individuals, appearing as ‘high sensitivity’ in roughly 30% of adults [9]. High sensitivity is mediated via neural and genetic factors [10–12] that are thought to predispose individuals to adverse conditions (such as stress, poor health and disorders) in harsh environments [13], as well as bestowing benefits in supportive ones [7,14,15].

According to SPS theory, the trait is characterized by greater depth of processing, cognizance of subtleties in the environment, being easily overstimulated, having stronger emotional responses (both positive and negative), and empathy to others' affective cues [6,10,11,16–18]. Found in over 100 other species [11], including primates [18], SPS is thought to be a survival strategy that may facilitate behaviours to garner resources, provide responsive care to others, and avoid threats through careful observation of each situation and then comparing it to past observations. However, high sensitivity is not adapted by all organisms within a given species because it has cognitive and physiological costs. Furthermore, if all organisms were equally sensitive, there would be no advantage to it. Hence, high sensitivity is thought to be ‘negative frequency dependent’, found only in a minority of individuals within a given species.

Similarly, differential susceptibility (DS) and biological sensitivity to context (BSC) theories propose that individuals vary in their degree of sensitivity, and thus highly sensitive persons are more susceptible both to the detrimental effects of harsh environments, and the benefits of positive and nurturing ones [6,14,15,19–21].

In recent times, there has been increased attention to sensory processing disorders and Autism Spectrum Disorders (ASD), particularly in children, which are also characterized by hyper- or hypo-sensitivity to tactile, auditory and visual stimuli [22]. However, in contrast to SPS, individuals with ASD typically show social communicative difficulties, impaired empathy, restricted interests and repetitive behaviours [23,24]. In children with ASD, social deficits—such as difficulty in making eye contact, facial recognition, responding to others' emotional cues and reciprocating intentions—appear as early as a few months to 2–3 years of age [25–32]. Indeed, a large body of work, including diagnostic tools, suggests that social impairments in ASD are largely mediated by deficient responsivity of neural structures involved in emotion, facial processing, empathy and reflective thinking [33–37]. By contrast, fMRI research on SPS has tended to show prominent brain activation of regions that are implicated in empathy, social processing and reflective thinking [10,20].

Somewhat similarly, post-traumatic stress disorder (PTSD) is also characterized by maladaptive responses to triggering stimuli that are misperceived as harmful due to their similarity to stimuli present during the original trauma. This also leads to a generalized vigilance for threats and difficulty in properly evaluating sensory stimuli. These impairments manifest in one of two ways: as hyperarousal to stimuli or as dissociation that may appear as under-response to the triggering stimulus [38]. These disruptions often result in the inability to properly integrate memories and a fixation on the past [39]. Correspondingly, brain imaging studies suggest that PTSD is associated with dysregulated response in neural regions that process emotion, attention, memory and self-control [40–44]. These studies suggest there is a general increase in emotional and vigilance-related brain activation in the amygdala; and diminished self-control, shown as PFC deactivation [45]; while remediation from PTSD shows the reverse [44]. Interestingly, some PTSD studies have shown diminished activation in sensory and temporal areas, supporting ideas that PTSD may also manifest as dissociation or lack of present-moment awareness [46].

Schizophrenia (SZ) and other psychosis-related disorders are also characterized by the inability to inhibit irrelevant information, from stimuli, along with difficulties in social cognition and interactions, which are mediated by impaired theory of mind, emotion-processing, and agency judgments [33]. SZ also manifests as hyper-distractibility and marked deficits in working memory. Its active symptoms include auditory and/or visual hallucinations without corresponding sources in the external world [47]. As such, neuroimaging studies that have compared SZ patients' (versus healthy subjects) response to emotional stimuli (facial expressions) indicate abnormal activation of brain regions involved in emotion, memory, attention, empathy, multisensory integration, reflective thinking and self-control [33,48–53]. Clearly, these various disorders have overlapping symptoms, as ASD is also characterized by marked impairments in empathic and social processes and behaviours [29]; and all seem to show maladaptive responsiveness to incoming emotional stimuli. They are also well known and include a broad symptom of categories, therefore, motivating our selection for comparisons.

As evidence suggests that there are behavioural, conceptual and perceptual differences across SPS, ASD, PTSD and SZ—typically with regard to empathy, social interactions and emotional responsivity to salient stimuli—we conducted a systematic review of functional MRI studies examining response to emotional and perceptual tasks across these fields to better understand the similarities and differences, as well as underlying neural correlates, for these conditions that involve ‘sensitivity’ to stimuli. We based this review on fMRI literature even though there are only four fMRI studies of SPS. However, we believe the comparison these allow, although only preliminary, will be useful because of the clear differences across these conditions.

## 2. Methods

First, we retrieved the four fMRI studies of SPS, and meta-analyses and review papers investigating ASD, PTSD and SZ with fMRI that involved tasks similar to those in the SPS studies: for example, studies examining responses to affective or social stimuli, emotion recognition or perceptual tasks. For ASD, PTSD and SZ, we focused on systematic reviews and meta-analyses, as the body of work on these are abundant. However, we also reviewed two individual fMRI studies of ASD that examined neural response to positive social stimuli and familiar versus unfamiliar faces [10,11], which very directly aligned with a study examining empathy as a function of SPS [1]. Moreover, we only included studies of marked ASD and excluded milder forms of the disorder, such as High-Functioning Autism (HFA) to make for a strong comparison with SPS, as studies have tended to find differing results for samples low on Autism or with experimental paradigms that were not truly measuring empathy.

## 3. Results

This review included 27 peer-reviewed fMRI research articles, meta-analyses and review papers examining neural responsiveness to emotional, social or salient stimuli: four regarding SPS, eight ASD, nine SZ and seven PTSD (one meta-analysis reported on social cognition studies for both SZ and ASD [33]). Brain region results and sample characteristics for SPS and ASD studies are shown in the electronic supplementary material, tables S1 and S2, respectively.

We compared patterns of activation and deactivation for SPS, ASD, SZ and PTSD studies. Common activations across all four were shown in the precentral gyrus. Activations unique to SPS were shown in neural structures associated with reward processing (VTA and SN, for positive stimuli only), physiological homeostasis and pain-control (hypothalamus and PAG); self-other processing and empathy (IFG and insula), awareness and reflective thinking (TPJ) and self-control (PFC)—while they showed deactivations or lack of results for ASD, PTSD and SZ in the context of emotional, social and perceptual tasks. However, SZ was dissimilar from SPS, ASD and PTSD in its showing deactivations in the caudate, thalamus, amygdala, cingulate/anterior cingulate cortex (ACC), superior frontal gyrus (SFG), precuneus, medial temporal gyrus and superior temporal lobe/gyrus (STL/STG)—while these areas were activated for SPS, ASD and PTSD (with some variability).

Comparison of SPS and ASD studies showed common neural activations in the caudate, thalamus, STG/STL, supramarginal gyrus and precuneus. However, SPS showed clear activations, whereas ASD showed deactivations, in the VTA/SN (for positive stimuli only); amygdala (emotion), hippocampus (memory), hypothalamus and PAG; as well as regions involved in empathy and self-other processing (insula, AI, IFG, FG); self-control and executive function (MFG and PFC); and the default mode network (DMN) including the temporal, TPJ, parietal and angular gyrus (AG).

## 4. Discussion

The present review highlights common and unique neural circuits reported for SPS as compared to ASD, PTSD and SZ–disorders that involve sensory issues and hyper- or hypo-responsiveness to stimuli. A common brain activation reported in the literature across these four conditions was the precentral gyrus; a primary site of motor command that is involved in conscious movement [54]. Common activations for SPS, ASD and PTSD (showing deactivation or lack of activation for SZ) were the caudate (reward processing), thalamo-cingulate circuit (attention) and areas of the DMN (SFG, precuneus and temporal areas) involved in reflective thinking, motor and cognitive control. These results highlight some neural structures that may coordinate hyper-sensitivity symptoms displayed in SPS, ASD and PTSD, and also suggests how SPS may be correlated with some of these conditions. For example, individuals with high (versus low) SPS may be more susceptible to PTSD and other adverse reactions following trauma exposure [55].

Neural activations that appeared for SPS, but showed deactivations or lack of activations for ASD, PTSD and SZ, were shown in regions that mediate reward (to positive stimuli only), hormonal balance, calm, empathy, self-reflective thinking and self-control (hypothalamus, PAG, IFG, insula, TPJ and PFC). These brain structures highlight some of the primary features that differentiate SPS from the disorders reviewed herein, such as enhanced conscientiousness, empathy and depth of processing [6]. Highly sensitive individuals do experience hyperarousal to some stimuli, such as when feeling empathy towards others' distress or in the presence of unusually loud noises, but these may be moderated in at least some individuals, if not most, by increased physiological calm and homeostasis, as well as cognitive and emotional control. This requires regulating unpleasant emotional states to optimize outcomes for the self and others as seen by prominent activation of the PFC, involved in self-regulation. SPS is characterized by a deep integration of information and intricate memory processing. These two neural signatures of SPS processing are facilitated when the organism remains calm in the presence of a stimulus while engaging other emotional, cognitive and sensory systems.

However, for ASD, PTSD and SZ, dysregulated responses in the hippocampus, insula and DMN are typical [52,56], disrupting memory and integration of information. Also in ASD, PTSD and SZ, decreased reward to positive stimuli plus a lack of empathy, calmness and self-control coincide with disruptions in social behaviours, as seemingly benign stimuli may be perceived as threats.

For ASD specifically, the reward, emotional and calming-inducing nature of social stimuli appear to be impaired, as manifested in diminished VTA, amygdala, hippocampal, hypothalamic and PAG activations. This results in lessened self-other processing and decreased empathy (deactivation of the IFG, FG and insula, and AG) that are cardinal features of ASD as confirmed by many studies [29,33]. We suggest that these differences in response to social stimuli for ASD (compared with SPS) may also be due to the diminished calm and reward inducing elements that are typically evoked for social/affective events in neuro-typical individuals. This suggests perhaps that a key divergence for SPS and ASD may be the extent to which individuals find social/emotional stimuli rewarding and are able to physiologically/hormonally and behaviourally respond adaptively to both positive and negative social stimuli.

The present review highlights some of the principal features of SPS, ASD, SZ and PTSD; and provides some key neural patterns that may serve to distinguish SPS from clinical disorders with sensory symptoms. They also help to clarify practical issues around SPS and neurodiversity perspectives on ASD which otherwise have suggested that all sensory issues are clear indications of ASDs. It is important to note that ASD is a spectrum showing varying degrees of symptoms with some individuals having HFA or Asperger’s. However, the present review only included studies of diagnosed ASD, and excluded studies in which the majority of subjects were HFA.

With only four fMRI studies involved in SPS, it is clear that more research is needed on this topic. Although this review may seem preliminary, it seemed warranted to begin to clarify how SPS is distinct from disorders that may seem related due to symptoms and hyperarousal to stimuli. However, as shown by fMRI studies, SPS is clearly different with respect to empathy, emotional responsiveness, self-reflection, physiological calm and self-regulation. Thus, this review may serve as a basis for clarification of diagnoses, practical questions, and further research and developments.

## 5. Conclusion

SPS is distinct from seemingly related clinical disorders—such as ASD, PTSD and SZ—that have overlapping symptoms such as sensitivity to the environment and hyper- or hypo-responsiveness to stimuli. Consistent with research suggesting that SPS is associated with greater empathy and awareness in response to social, emotional and perceptual tasks, we found that SPS differentially engages brain regions involved in reward processing (for positive stimuli), memory, physiological homeostasis, self-other processing and awareness. We suggest that adaptive SPS strategies involving empathy, awareness, calmness and physiological and cognitive self-control may serve a species by facilitating deep integration and memory for environmental and social information, which may ultimately foster survival, well-being and cooperation.

## Supplementary Material

Supplementary Tables

## Data accessibility

All data gathered for this review may be available upon request to the corresponding author.
