# Supplementary material for: The functional highly sensitive brain: a review of the brain circuits underlying sensory processing sensitivity and seemingly related disorders
Source: Philos Trans R Soc Lond B Biol Sci. 2018 Feb 26;373(1744):20170161. doi: 10.1098/rstb.2017.0161 (PMC5832686; doi:10.1098/rstb.2017.0161)
Supplement: Supplementary Tables [file rstb20170161supp1.docx]

**Supplementary Table 1.** Comparison of Major Regional Neural Activation Patterns Reported in fMRI studies of Sensory Processing Sensitivity, Autism Spectrum Disorder, Post-traumatic Stress Disorder and Schizophrenia

| **Brain Region** | **SPS** | **Ref** | **ASD** | **Ref** | **PTSD** | **Ref** | **SZ** | **Ref** |
| --- | --- | --- | --- | --- | --- | --- | --- | --- |
| **VTA/SN** | Activation | 1, 2 | NA |  | NA |  | NA |  |
| **Caudate** | Activation | 1, 2 | Activation | 6 | Activation  Deactivation | 13, 14  15 | Deactivation | 16 |
| **Thalamus** | Activation | 2 | Activation | 6 | Activation  Deactivation | 14, 15, 17  15, 18 | Deactivation | 7, 19 |
| **Hypothalamus** | Activation | 1 | Deactivation | 6 | Deactivation | 18 | NA |  |
| **PAG** | Activation | 1 |  | NA | NA |  | NA |  |
| **Hippocampus** | Activation | 1, 2 | Deactivation | 12 | Activation  Deactivation | 14 15, 17  18 | Deactivation | 16, 19, 20 |
| **Amygdala** | Activation | 1, 2 | Activation  Deactivation | 11  7, 9, 12 | Activation | 13, 14, 15, 17, 18, 21, 22 | Deactivation | 7, 16, 20 23, 24 |
| **Cingulate/ ACC** | Activation | 1, 2 | Activation  Deactivation | 9, 12  7, 12 | Activation  Deactivation | 13, 15, 17, 22  14, 15, 18 | Activation  Deactivation | 7  16, 20 |
| **Insula** | Activation | 1, 2 | Deactivation | 8, 12 | Activation  Deactivation | 14, 15, 18  13, 15, 17 | Deactivation | 23, 24 |
| **Anterior insula** | Activation | 2 | Deactivation | 8 | Activation | 14, 15, 21 | Deactivation | 20, 25, 26 |
| **IFG** | Activation | 2 |  | NA | Activation  Deactivation | 17  15, 18, 21, 22 | Deactivation | 7 |
| **MFG** | Activation | 3 | Deactivation | 7, 11, 12 | Activation | 13, 14, 15, 17 | Activation  Deactivation | 7  16, 20 |
| **SFG** | Activation | 2 | Activation  Deactivation | 12  12 | Activation | 14 | Deactivation | 20 |
| **Orbitofrontal cortex** | Deactivation | 2 | Activation | 11 | Activation  Deactivation | 14  15 | Deactivation | 20 |
| **PFC** | Activation | 1, 2, 4 | Deactivation | 7 | Activation  Deactivation | 14  13, 15, 18, 21, 22 | Deactivation | 7 |
| **Temporal/TPJ** | Activation | 1, 2, 3 | NA |  | Deactivation | 13 | Deactivation | 19, 20 |
| **MTG** | Activation | 1, 2, 3 | Activation  Deactivation | 12  7 | Activation  Deactivation | 14  13 | Activation  Deactivation | 7  16, 19, 20 |
| **STL/STG** | Activation | 1, 2, 3 |  | NA | Activation | 14 | Deactivation | 19, 20 |
| **FG/Occipital region** | Activation | 1, 3 | Deactivation | 5, 9, 11, 12 | Activation | 14, 15 | Activation  Deactivation | 16  7, 16, 19, 27 |
| **SMG** | Activation | 2, 3 | Activation | 12 | Deactivation | 15 | Deactivation | 19 |
| **Parietal lobe** | Activation | 1, 2, 3, 4 |  | NA | Activation | 17, 18 | Activation  Deactivation | 16  19, 20 |
| **Precuneus** | Activation | 1, 2, 3 | Activation | 6, 12 | Activation | 13, 14, 15, 18 | Deactivation | 19, 20 |
| **Angular gyrus** | Activation | 2 | Deactivation | 12 | Activation  Deactivation | 13  15 | NA |  |
| **PMA** | Activation | 2 | NA |  | Activation | 14 | NA |  |
| **SMA** | Activation | 2 | NA |  | Activation | 14 | Deactivation | 19 |
| **SI** | Activation | 2 | Deactivation | 7 | Activation | 14 | Activation | 7 |
| **Precentral Gyrus** | Activation | 2 | Deactivation | 7, | Activation  Deactivation | 13  15 | Activation  Deactivation | 16  19, 20 |
| **Postcentral Gyrus** | Activation | 2 | Activation  Deactivation | 12  7 | Activation  Deactivation | 15  13 | Deactivation | 19 |

**Abbreviations for Brain Regions.** ACC = anterior cingulate cortex. FG = fusiform gyrus (also known as fusiform face area, FFA). IFG = inferior frontal gyrus. PAG = periaqueductal gray. MFG = middle frontal gyrus. PFC = prefrontal cortex. PMA = premotor area. MTG = middle temporal gyrus.
SFG = superior frontal gyrus. SI = primary sensory areas. SMA = supplementary motor area. SMG = supramarginal gyrus. STL = superior temporal lobe. STG = superior temporal gyrus. SN = substantia nigra. TPJ = temporoparietal junction. VTA = ventral tegmental area.

| Supplementary Table 2. fMRI studies of Sensory Processing Sensitivity and Autism Spectrum Disorder examining brain responsivity to emotional stimuli | | | | | | | |
| --- | --- | --- | --- | --- | --- | --- | --- |
| SPS Article/ | SPS | | Control | | Scale | Task | Major Results |
| Reference # | **n** | **M Age** | **n** | **M Age** |  |  |  |
|  | **Gender (n)** | | **Gender (n)** | |  |  |  |
|  | **Other** | | **Other** | |  |  |  |
| 1. Acevedo et al. (2017) | 14 | 19 | none | | HSP Scale, Neuroticism, Quality of Childhood Parenting (QCP) | Participants viewed positive, negative and neutral IAPS images while undergoing fMRI. | -SPS (and its interaction with QCP) showed significant activation in areas associated with emotional memory, physiological homeostasis, awareness, and reflective thinking (hippocampus, entorhinal area, hypothalamus; temporal and parietal areas).  - SPS was positively correlated with activation of areas involved in reward, calm, and self/other processing (i.e., VTA, PAG, insula, and IFG) for positive stimuli only.  - For negative stimuli, SPS x QCP showed prominent activation in the PFC without reward (VTA) diminishment. |
|  | m/f=0/14 | |  |  |  |  |  |
|  |  | |  |  |  |  |  |
| 2. Acevedo et al. (2014) | 18 | 27.50 | none | | HSP Scale,  Neuroticism | Participants viewed photos of their romantic partners and strangers showing happy, sad or neutral facial expressions at baseline and after one year while undergoing fMRI. | - SPS showed significant neural correlations with happy and sad expressions in the, insula, IFG, cingulate, MTG, and premotor areas that are implicated in awareness, sensory integration, reflective thinking empathy and action planning. |
|  | m/f=8/10 | |  |  |  |  |  |
|  | In established romantic relationships (M=4.3 yrs.) | |  |  |  |  |  |
| 3. Jagiellowicz et al. (2010) | 16 | 21.6 | none | | HSP Scale,  Neuroticism,  Introversion | Participants viewed photos of natural and man-made scenes with major or minor alterations. | -SPS showed significant activation in areas involved in visual processing (i.e., claustrum, occipitotemporal regions), even after controlling for Neuroticism and Introversion in response to viewing minor stimuli changes. |
|  | m/f=8/8 | |  |  |  |  |  |
|  |  | |  |  |  |  |  |
| 4. Aron et al. (2010) | 20 | 18-26 | none | | HSP Scale | Participants viewed line-length while attending to versus ignoring context. | - SPS showed significant activation in frontal and parietal regions associated with attention and working memory for the culturally non-preferred task in both groups.  -SPS showed inverse associations: high SPS individuals demonstrated little cultural difference and vice versa. |
|  | m/f=9/11 | |  |  |  |  |  |
|  | 10 Asians  10 European-Americans | |  |  |  |  |  |

| ASD Article | ASD Group | | | | Control | | | | Scale | Task | Major Results |
| --- | --- | --- | --- | --- | --- | --- | --- | --- | --- | --- | --- |
| Reference # | **n** | **M Age** | | | **n** | **M Age** | | |  |  |  |
|  | **Gender (n)** | | | | **Gender (n)** | | | |  |  |  |
|  | **Other** | | | | **Other** | | | |  |  |  |
| *Meta-analyses of fMRI Studies* |  | | | |  | | | |  |  |  |
| 5. Nickl-Jockschat et al. (2015)  (N=14) | 164 | 11.3-37 | | | 165 | | 11.8-35.3 | | -ADOS  -ADI-R  -DSM-IV  -ICD-10 | Face processing in ASD versus controls. | -ASD studies showed one cluster of decreased activation in the FG during face-viewing.  -Both task and resting state showed functional connectivity of FG with temporo-occipital and lateral occipital cortex, inferior frontal and parietal cortex, thalamus and amygdala.  - Results suggest a functionally and structurally disturbed network of occipital regions related primarily to face- (but perhaps also language) processing, interact with inferior frontal as well as limbic regions and may mediate aberrant face-processing in ASD. |
|  | m/f=158/6 | | | | m/f=158/7 | | | |  |  |  |
|  |  | | | |  | | | |  |  |  |
| 6. Aoki, Cortese, Tansella (2015)  (N=13) | 226 | | 9-37 | | 251 | | | 9.2-28.6 | “established diagnostic tools” | Emotional face processing (“emotional-face vs. non-emotional-face” or “emotional-face vs non-face”). | **-**ASD showed activation in the caudate, thalamus, cingulate, and precuneus; and deactivation in the hypothalamus during emotional-face processing. |
|  |  | | | |  | | | |  |  |  |
|  | 226 HFA | | | |  | | | |  |  |  |
| 7. Sugranyes et al. (2011)  (N=33: ASD and SZ) | 482  146 ASD  SZ 336 | | | ASD: 32 SZ: 31 | 492 | | | Age-match to SZ and ASD | -ADOS  -ADI-R  -AQ  -ADI-R  -DSM-IV  -ICD-10 | Facial emotion recognition, Theory of Mind (ToM), judgment of others’ state (from eyes); eye gaze shift; empathy; corruption; cooperation; deceit  -Self-face recognition  -Facial expression imitation  -Empathic story completion  -Self/other labeling  -Intentional story completion | -ASD and SZ showed deactivation in the mPFC relative to controls;  but more pronounced in ASD.  -For ASD, deactivation of the amygdala was seen during tasks, and in SZ during facial emotion recognition.  -ASD showed activation in the STS during affect-processing, but both ASD and SZ showed deactivation in the STS for ToM tasks.  -Overall, ASD showed deactivation in somatosensory regions, but showed activation in SZ). |
|  | ASD: 100% males;  SZ: 74.93% males | | | |  | | | |  |  |  |
|  |  | | | |  | | | |  |  |  |

| 8. Di Martino et al. (2009)  (N=39 studies:  24 social, 15 non-social) | 479 | 12.7-28.2 | 243 | 12.7-27.7 | -PDD  -NOS | -24 social: ToM, face processing, and emotional processing.  -15 non-social: spatial attention, interference control, working memory, and motor control. | -Social tasks: ASD (versus controls) showed significant deactivation of the amygdala, parahippocampal gyrus, thalamus, FG (especially for faces), IFG, AI, mPFC, and pgACC, AC, PCC, lingual gyrus, occipital gyrus. Showed greater activation in temporal areas, precentral gyrus, postcentral gyrus, |
| --- | --- | --- | --- | --- | --- | --- | --- |
|  |  | |  | |  |  |  |
|  |  | |  | |  |  |  |
| *Reviews of fMRI Studies* |  | |  | |  |  |  |
| 9. *Harms, Martin, Wallace (2010) |  | |  | | -PDD  -NOS  -DSM-V  -DSM-IV | Abnormal eye gaze patterns, delayed event-related potential, emotion-processing circuitry. | Neuroimaging studies showed:  -Activation in limbic regions (e.g. amygdala).  -Deactivation in medial-frontal and orbitofrontal cortices, IFG, FG , MTG and the amygdala.  -Activation in the vmPFC, STS and ACC.  Electrophysiological studies: weaker/slower responses in frontal, fusiform, and visual cortices; slower/larger responses in parietal somatosensory cortices. |
| 10. Schultz (2005) |  | |  | | -DSM  -IV  -PPD  -NOS | -Face perception and detection of emotionally salient percepts | -ASD showed deactivation of the FG, amygdala during face perceptual tasks; -heuristic model of the pathophysiology of autism: early deficits in amygdala, with a cascading influence on the development of cortical areas that mediate social perception, such as FG and temporal lobe. |
| *Individual Studies* | | |  | |  |  |  |
| 11. Dalton et al. (2005) | Study I | | Study I | | -ADI-R  -DSM-IV | -Emotional and neutral faces.  -Familiar and unfamiliar faces | -ASD showed strong positive correlation between time spent fixating the eyes and activation of FG and amygdala.  -In ASD, variation in eye fixation showed strong positive correlation with amygdala activation. |
|  | 14 | 15.9 | 12 | 17.1 |  |  |  |
|  | m/f=14/0 | | m/f=12/0 | |  |  |  |
|  |  | |  | |  |  |  |
|  | Study II | | Study II | |  |  |  |
|  | 16 | 14.5 | 16 | 14.5 |  |  |  |
|  | m/f=16/0 | | m/f=16/0 | |  |  |  |
|  | Autism & ASP | |  | |  |  |  |
| 12. Pierce, Haist, Sedaghat, Courchesne (2004) | 7 | 27.1 | 9 | 16-40 | -ADOS  -ADI  -ADI-R  -DSM-IV | Familiar female- vs stranger female-faces | -ASD showed right hemisphere dominance to both types of faces.  -ASD showed increased FG activity to familiar faces.  -Both groups showed fusiform face activation to both types of faces.  -Control group showed additional activation to familiar faces in PCC, amygdala and medial frontal lobes, including the anterior cingulate. ASD showed a similar, but more limited, network. |
|  | m/f=7/0 | |  | |  |  |  |
|  |  | |  | |  |  |  |

**Abbreviations for Brain Regions.** ACC = anterior cingulate cortex. FG = fusiform gyrus . IFG = inferior frontal gyrus. PAG = periaqueductal gray.
PCC = posterior cingulate cortex. pgACC = pregenual anterior cingulate cortex. MFG = middle frontal gyrus. mPFC = medial prefrontal cortex. MTG = middle temporal gyrus. SN = substantia nigra. STG = superior temporal gyrus. STS = superior temporal sulcus. vmPFC = ventromedial prefrontal cortex. VTA = ventral tegmental area. **Other Abbreviations.** ADI = autism diagnostic interview. ADI-R = autism diagnostic interview-revised. ADOS = autism diagnostic observation schedule. AQ = autism spectrum quotient. ASSQ = autism spectrum screening questionnaire. DSM = diagnostic and statistical manual of mental disorders. ICD-10 = international classification of diseases-tenth edition. SRS = social responsiveness scale. **Other terms.**  ASD = autism spectrum disorder. ASP = Asperger’s Syndrome. HFA = high functioning autistic. HSP = highly sensitive person. n = sample size. N = number of studies in meta-analysis or review studies. SZ = schizophrenia. SPS = sensory processing sensitivity.

**References for Tables 1 and 2**

1. Acevedo B, Jagiellowicz J, Aron E, Marhenke R, Aron A. 2017. Sensory Processing Sensitivity and Childhood Quality’s Effects on the Human Brain’s Emotional Response.
2. Acevedo BP, Aron EN, Aron A, Sangster MD, Collins N, Brown LL. 2014. The highly sensitive brain: an fMRI study of sensory processing sensitivity and response to others' emotions. *Brain Behav.* 4(4),580-94. (doi:10.1002/brb3.242)
3. Jagiellowicz J, Xu X, Aron A, Aron E, Cao G, Feng T, Weng X. 2011. The trait of sensory processing sensitivity and neural responses to changes in visual scenes. *Soc Cogn Affect Neurosci.* 6(1), 38-47. (doi: 10.1093/scan/nsq028)
4. Aron A, Ketay S, Hedden T, Aron EN, Rose Markus H, Gabrieli JD. 2010. Temperament trait of sensory processing sensitivity moderates cultural differences in neural response. *Soc Cogn Affect Neurosci.* 5(2-3), 219-226. (doi: 10.1093/scan/nsq028)
5. Nickl-Jockschat T, Rottschy C, Thommes J, Schneider F, Laird AR, Fox PT, Eickhoff SB. 2015. Neural networks related to dysfunctional face processing in autism spectrum disorder. *Brain Struct Funct.* 220(4):2355-2371. (doi:10.1007/s00429-014-0791-z)
6. Aoki Y, Cortese S, Tansella M. 2015. Neural bases of atypical emotional face processing in autism: A meta-analysis of fMRI studies. *World J Biol Psychiatry.* 16(5), 291-300. (doi:10.3109/15622975.2014.957719)
7. Sugranyes G, Kyriakopoulos M, Corrigall R, Taylor E, Frangou S. 2011. Autism spectrum disorders and schizophrenia: meta-analysis of the neural correlates of social cognition. *PloS one*. 6(10),e25322. (doi: https://doi.org/10.1371/journal.pone.0025322)
8. Di Martino A, Ross K, Uddin LQ, Sklar AB, Castellanos FX, Milham MP. 2009. Functional brain correlates of social and nonsocial processes in autism spectrum disorders: an activation likelihood estimation meta-analysis. *Biol Psychiatry*. 65(1),63-74. (doi:10.1016/j.biopsych.2008.09.022)
9. Harms MB, Martin A, Wallace GL. 2010. Facial emotion recognition in autism spectrum disorders: a review of behavioral and neuroimaging studies. *Neuropsychol Rev.* 20(3),290-322. (doi:10.1007/s11065-010-9138-6)
10. Schultz RT. 2005. Developmental deficits in social perception in autism: the role of the amygdala and fusiform face area. *Int J Dev Neurosci*. 23(2-3),125-41. (doi:10.1016/j.ijdevneu.2004.12.012)
11. Dalton KM, Nacewicz BM, Johnstone T, Schaefer HS, Gernsbacher MA, Goldsmith HH, Alexander AL, Davidson RJ. 2005. Gaze fixation and the neural circuitry of face processing in autism. *Nat Neurosci.* 8(4),519-526. (doi:10.1038/nn1421)
12. Pierce K, Haist F, Sedaghat F, Courchesne E. 2004. The brain response to personally familiar faces in autism: findings of fusiform activity and beyond. *Brain.* 127(Pt 12), 2703-2716. (doi:10.1093/brain/awh289)
13. Sartory G, Cwik J, Knuppertz H, Schürholt B, Lebens M, Seitz RJ, Schulze R. In search of the trauma memory: a meta-analysis of functional neuroimaging studies of symptom provocation in posttraumatic stress disorder (PTSD). *PLoS One*. 2013,8(3),e58150. (doi:10.1371/journal.pone.0058150)
14. Stark EA, Parsons CE, Van Hartavelt TJ, Charquero-Ballester M, McMAnners H, Ehlers A, Stein A, Kringelbach ML. 2015. Post-traumatic stress influences the brain even in the absence of symptoms: A systematic, quantitative meta-analysis of neuroimaging studies. *Neurosci Biobehav.* 56, 207-21. (doi: 10.1016/j.neubiorev.2015.07.007)
15. Patel R, Spreng RN, Shin LM, Girard TA. 2012. Neurocircuitry models of posttraumatic stress disorder and beyond: a meta-analysis of functional neuroimaging studies. *Neurosci Biobehav Rev.* 36(9),2130-42. (doi:10.1016/j.neubiorev.2012.06.003)
16. Taylor SF, Kang J, Brege IS, Tso IF, Hosanagar A, Johnson TD. 2012. Meta-analysis of functional neuroimaging studies of emotion perception and experience in schizophrenia. *Biol Psychiatry*. 71(2),136-45. (doi:10.1016/j.biopsych.2011.09.007)
17. Boccia M, D'Amico S, Bianchini F, Marano A, Giannini AM, Piccardi L. 2016. Different neural modifications underpin PTSD after different traumatic events: an fMRI meta-analytic study. *Brain Imaging Behav.* 10(1), 226-237. (doi:10.1007/s11682-015-9387-3)
18. Etkin A, Wager TD. Functional neuroimaging of anxiety: a meta-analysis of emotional processing in PTSD, social anxiety disorder, and specific phobia. *Am J Psychiatry*. 2007,164(10),1476-88. (doi:10.1176/appi.ajp.2007.07030504)
19. Goghari VM, Sanford N, Spilka MJ, Woodward TS. 2017. Task-Related Functional Connectivity Analysis of Emotion Discrimination in a Family Study of Schizophrenia. *Schizophr Bull*. (doi:10.1093/schbul/sbx004)
20. Laurens KR, Kiehl KA, Ngan ETC, Liddle PF. 2005. Attention orienting dysfunction during salient novel stimulus processing in schizophrenia. *Schizophr Res.* 75(2-3), 159-71. (doi: http://dx.doi.org/10.1016/j.schres.2004.12.010)
21. Hayes JP, Hayes SM, Mikedis AM. 2012. Quantitative meta-analysis of neural activity in posttraumatic stress disorder. *Biol Mood Anxiety Disord*. 2,9. (doi:10.1186/2045-5380-2-9)
22. Koenigs M, Grafman J. 2009. Posttraumatic stress disorder: the role of medial prefrontal cortex and amygdala. *Neuroscientist*. 15(5),540-8. (doi:10.1177/1073858409333072)
23. Seiferth NY, Pauly K, Kellermann T, Shah NJ, Ott G, Herpertz-Dahlmann B, Kircher T, Schneider F, Habel U. 2009. Neuronal correlates of facial emotion discrimination in early onset schizophrenia. *Neuropsychopharmacology.* 34(2),477-487. (doi:10.1038/npp.2008.93)
24. Williams LM, Das P, Liddell BJ, Olivieri G, Peduto AS, David AS, Gordon E, Harris AW. 2007. Fronto-limbic and autonomic disjunctions to negative emotion distinguish schizophrenia subtypes. *Psychiatry Res.* 155(1), 29-44. (doi:10.1016/j.pscychresns.2006.12.018)
25. Wylie KP, Tregellas JR. 2010. The role of the insula in schizophrenia. *Schizophr Res*. 123(2-3),93-104. (doi:10.1016/j.schres.2010.08.027)
26. Phillips ML, Williams L, Senior C, Bullmore ET, Brammer MJ, Andrew C, Williams SC, David AS. 1999. A differential neural response to threatening and non-threatening negative facial expressions in paranoid and non- eparanoid schizophrenics. *Psychiatry Res.* 92(1),11-31. (doi: 10.1016/S0925-4927(99)00031-1)
27. Cao H, Bertolino A, Walter H, et al. 2016. Altered Functional Subnetwork During Emotional Face Processing: A Potential Intermediate Phenotype for Schizophrenia. *JAMA Psychiatry.* 73(6),598-605. (doi:10.1001/jamapsychiatry.2016.0161)
